# Omentoplasty Versus Tube Drainage After Conservative Surgery for Hepatic Cystic Echinococcosis at High Risk of Biliary Complications: A Prospective Comparative Study

**DOI:** 10.3390/jcm15218299

**Published:** 2026-10-25

**Authors:** Bilal Aydın, Metin Yıldız, Nurhak Aksungur, Necip Altundaş, Salih Kara, Rifat Burak Ergül, Gürkan Öztürk

**Affiliations:** 1 Department of General Surgery, Faculty of Medicine, Ataturk University, Erzurum 25240, Türkiye; 2 Department of General Surgery, Mus State Hospital, Mus 49200, Türkiye; 3 Department of Urology, Mus State Hospital, Mus 49200, Türkiye

**Keywords:** hepatic cystic echinococcosis, conservative surgery, omentoplasty, tube drainage, residual cavity, bile leakage

## Abstract

**Background/Objectives:** Residual cavity complications contribute to morbidity after conservative surgery for hepatic cystic echinococcosis (HCE). We compared omentoplasty and tube drainage in patients with HCE at high risk of biliary complications. **Methods:** This prospective, single-center, nonrandomized study included 60 patients allocated alternately to omentoplasty or tube drainage (30 per group). The primary outcome was bile leakage and/or residual cavity complications within 30 days after surgery. Exploratory Firth logistic regression adjusted for cyst diameter and preoperative cholangitis. Holm correction was applied to five secondary outcomes. **Results:** The primary outcome occurred in 6/30 (20.0%) versus 16/30 (53.3%) patients (risk ratio 0.375; 95% confidence interval [CI] 0.170–0.826; *p* = 0.015). Adjusted odds were higher with tube drainage (odds ratio 5.25; 95% CI 1.51–22.72; *p* = 0.008). This association persisted after additional adjustment for preoperative endoscopic retrograde cholangiopancreatography. Surgical site infection (3.3% vs. 30.0%) and percutaneous intervention (0% vs. 26.7%) were less frequent with omentoplasty (Holm-adjusted *p* = 0.049 and 0.023, respectively). The operative-time difference was not significant after Holm adjustment. Bile leakage occurred in 20.0% versus 36.7% (*p* = 0.252). Mean follow-up was 68.8 ± 5.8 months; all patients completed at least 60 months, with no loss to follow-up. Recurrence occurred in 3.3% versus 13.3% (*p* = 0.353). **Conclusions:** Omentoplasty was associated with a lower incidence of the composite outcome, including after adjustment. These findings support its use in suitable patients and warrant confirmation in larger randomized studies.

## 1. Introduction

Hepatic cystic echinococcosis (HCE) is a zoonotic disease caused by parasites of the *Echinococcus granulosus* sensu lato complex and is associated with substantial morbidity and economic losses, particularly in endemic regions [1]. Türkiye remains an endemic country, where cystic echinococcosis continues to represent an important public health concern [2]. The liver is the most frequently affected organ. Treatment selection depends on cyst stage, size, and location, the presence of complications, and the patient’s clinical characteristics. Management options include medical therapy, percutaneous interventions, and surgery, whereas imaging surveillance is appropriate for uncomplicated inactive cysts. Surgery remains an important treatment option in patients with complications such as infection, rupture, or communication with the biliary tract [1,2,3].

Both radical and conservative approaches are used in the surgical treatment of HCE. Although lower rates of recurrence and residual cavity-related complications have been reported after radical surgery, its feasibility depends on cyst anatomy, the patient’s clinical condition, and surgical expertise. Conservative surgery remains widely used because it preserves liver parenchyma and can be performed in different anatomical settings [2].

The residual cavity is an important source of postoperative morbidity after conservative surgery. Bile leakage, cavity infection, and fluid collection may prolong hospitalization and necessitate additional interventions [4,5]. Residual cavity management is therefore an important component of surgical treatment. External tube drainage and omentoplasty are among the principal techniques used for this purpose. Omentoplasty fills the cavity with vascularized omental tissue, while the lymphatic and immunological properties of the omentum may also support healing [6]. Comparative studies and a meta-analysis specific to HCE have reported findings favoring omentoplasty, particularly for reducing deep abdominal infections [4,7,8]. In the liver surgery subgroup of a broader meta-analysis encompassing various surgical procedures, omentoplasty was also associated with lower risks of overall complications and postoperative infection [9].

However, the applicability of these findings to patients with HCE at high risk of biliary complications requires further evaluation. Previous studies differ in patient selection and cyst characteristics; for example, infected cysts were excluded from a prospective randomized comparison [5]. The association of cyst contents and complication status with postoperative outcomes highlights the importance of accounting for these clinical differences [10]. The Turkish HPB Surgery Association consensus report recommends favoring omentoplasty during conservative surgery in patients at risk of biliary fistula while acknowledging the low level of supporting evidence [2]. This recommendation highlights the importance of further evaluating the clinical outcomes of omentoplasty in patients at high risk of biliary complications. Further prospective comparisons addressing both early postoperative outcomes and long-term recurrence in this high-risk population are therefore warranted.

This prospective comparative study aimed to compare omentoplasty and tube drainage for residual cavity management after conservative surgery in a selected cohort of patients with HCE at high risk of biliary complications. This cohort included patients with existing or previously treated cyst-related complications, as well as patients considered at high risk on the basis of their cyst characteristics. The primary outcome was a composite of postoperative bile leakage or a residual cavity-related complication. Other early postoperative clinical outcomes and long-term recurrence were evaluated as secondary outcomes.

## 2. Materials and Methods

### 2.1. Study Design, Patient Selection, and Group Allocation

This prospective, single-center, nonrandomized comparative study included patients treated at the Department of General Surgery, Faculty of Medicine, Atatürk University, between March 2020 and May 2021. The study commenced following institutional ethics committee approval dated 27 February 2020 (meeting no. 2, decision no. 2). Written informed consent was obtained from all participants. The study was retrospectively registered at ClinicalTrials.gov under the protocol identifier ATAUNI-HCE-OPTD-2020 and registration number NCT07793448.

An a priori sample size calculation was performed using G*Power (version 3.1; Heinrich Heine University, Düsseldorf, Germany) [11]. Given the limited evidence directly comparing these approaches in this high-risk population, the expected incidence of the primary composite outcome was estimated before recruitment on the basis of our department’s previous clinical experience and clinical judgment as 10% in the omentoplasty group and 45% in the tube drainage group. Using a two-sided Fisher’s exact test, a significance level of 0.05, 80% power, and a 1:1 allocation ratio, the minimum required sample size was calculated as 29 patients per group. The study was completed with 60 patients, with 30 in each group.

Hepatic cystic echinococcosis (HCE) was assessed using ultrasonography (US) and contrast-enhanced computed tomography (CT). Cyst-related complications considered during clinical assessment included an infected cyst, cyst abscess, biliary involvement, intrabiliary rupture, cholangitis, spontaneous or traumatic rupture, or another complication requiring surgical treatment [1,2,3]. Existing or previously treated cyst-related complications were distinguished from features indicating an increased risk of biliary communication or postoperative biliary complications.

The study was designed to evaluate patients with HCE at high risk of biliary complications who were scheduled for open conservative surgery. This population included patients with existing or previously treated cyst-related complications, as well as patients considered at high risk on the basis of their cyst characteristics. Accordingly, patients were required to have at least one of the following features: a cyst diameter ≥ 8 cm; a cyst located in the central hepatic segments (IVa, IVb, V, or VIII); intrahepatic or extrahepatic bile duct dilatation on preoperative imaging; or a history of presentation with cholangitis or another biliary problem. Infected cyst contents and an abscess within the cyst cavity were also considered during patient selection [10]. Large cyst diameter and central location were regarded as risk indicators and did not, in themselves, establish the presence of a cyst-related complication.

Patients aged 18–80 years who met the eligibility criteria and provided written informed consent were included. Exclusion criteria were failure to meet the specified high-risk eligibility criteria, suspected alveolar echinococcosis, refusal to participate, and radical surgery or a primary percutaneous intervention as the definitive treatment for HCE during the study.

Patients scheduled for laparoscopic surgery were not enrolled and were managed according to routine clinical practice, outside the study allocation sequence. Laparoscopic HCE surgery was available at the center and was more frequently selected for patients considered to have a lower likelihood of cystobiliary communication.

**Figure 1:**
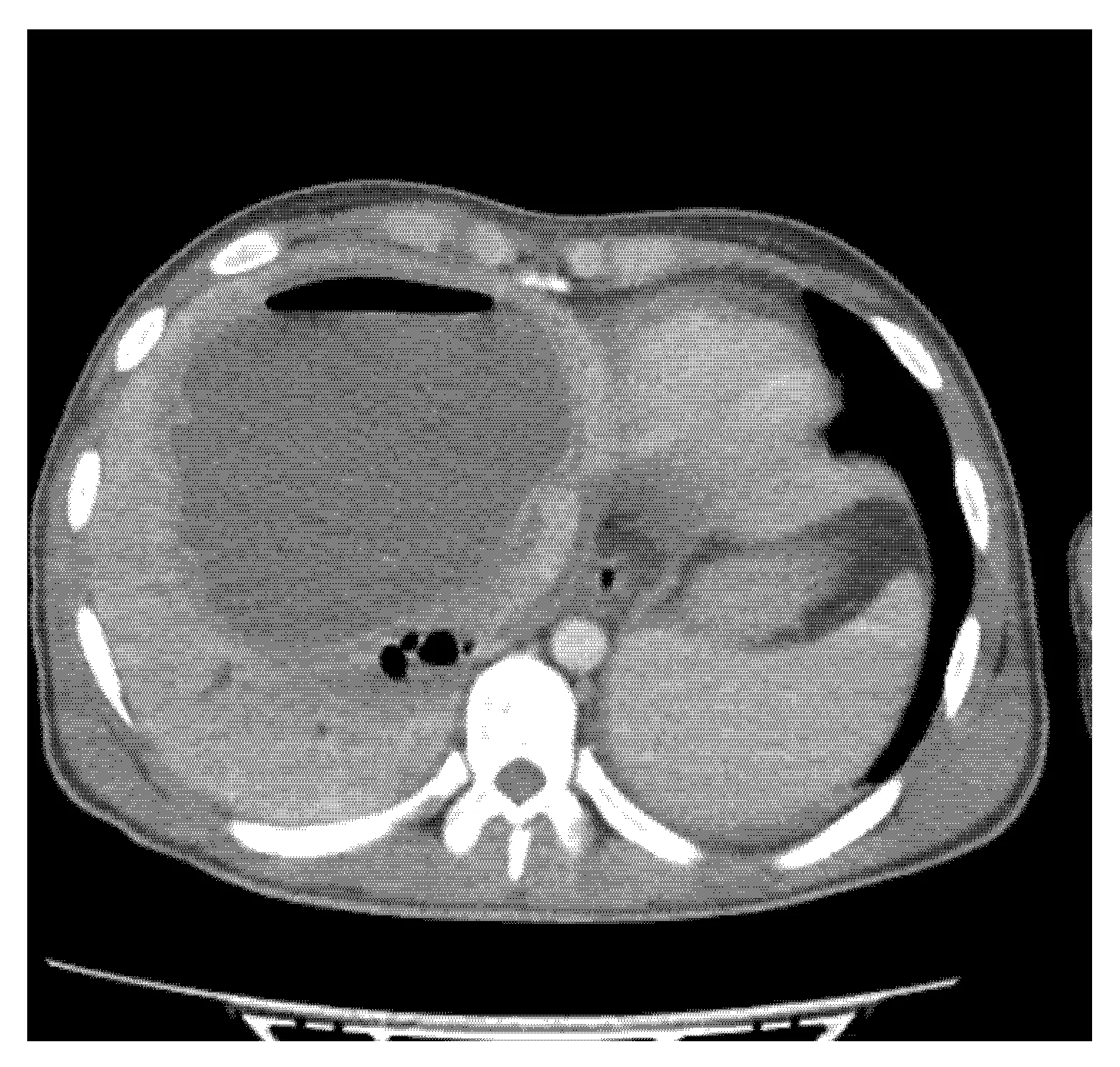
Preoperative contrast-enhanced axial CT image of a patient with complicated HCE who developed an intracystic abscess after PAIR and underwent conservative surgery. An air–fluid level is visible within the cyst cavity.

Previous puncture, aspiration, injection, and reaspiration (PAIR) was not, in itself, an exclusion criterion. Patients requiring conservative surgery following unsuccessful PAIR or abscess formation after PAIR were included if they met the remaining eligibility criteria. A preoperative CT image of a representative patient who developed an intracystic abscess after PAIR is presented in Figure 1.

Patients were allocated alternately to omentoplasty or tube drainage in a 1:1 ratio according to a sequence established before recruitment began. The allocation sequence was maintained and implemented by the study coordinator. Assignment took place after written informed consent had been obtained and the operation date had been scheduled. This approach was adopted to maintain numerical balance between the groups and to limit the influence of surgeon preference related to cyst location on the choice of residual cavity management. The assigned residual cavity management strategy was communicated by the study coordinator, in addition to providing the patient’s file, to the surgical team on the morning of surgery. The surgical team was not involved in the patient admission or informed consent processes. Allocation followed a nonrandom alternating sequence.

### 2.2. Perioperative Management, Outcome Measures, and Follow-Up

All patients underwent preoperative clinical assessment, US, contrast-enhanced CT, and serum biochemical testing. Age, sex, comorbidities, American Society of Anesthesiologists (ASA) physical status, presenting complaint, cyst number, cyst size and location, and preoperative interventions were recorded. Biochemical parameters evaluated included aspartate aminotransferase, alanine aminotransferase, alkaline phosphatase, and gamma-glutamyl transferase. Cysts were staged according to the World Health Organization Informal Working Group on Echinococcosis (WHO-IWGE) classification [3]. In patients with multiple cysts, size, location, and stage were recorded for the dominant cyst, while cyst number was documented separately. Segmental location was recorded according to the hepatic segment in which the cyst was predominantly located. This designation did not imply confinement to a single segment; extension into adjacent segments was not separately coded.

Albendazole was administered at 10 mg/kg/day for 20 days before surgery. Patients with suspected cholangitis or biliary obstruction underwent magnetic resonance cholangiopancreatography or endoscopic ultrasonography as clinically indicated. Endoscopic retrograde cholangiopancreatography (ERCP) was performed when therapeutic intervention was required. Patients presenting with cholangitis underwent treatment of the underlying biliary problem and clinical stabilization before surgery. The presenting complaint was recorded to reflect the initial clinical presentation before these interventions.

All operations were performed using an open approach by the same hepatopancreatobiliary surgical team. The abdominal cavity was accessed through a midline laparotomy or a modified J-shaped incision. Cystotomy was performed, the cyst contents were evacuated, and the contents were assessed for the presence of bile. No scolicidal agent was instilled into the cavity when bile-stained contents were identified. In the absence of bile staining, the residual cavity was irrigated with a 0.1% cetrimide–chlorhexidine solution.

The cavity was carefully inspected, and visible biliary openings suitable for closure were sutured with 4/0 polypropylene. Particular caution was exercised when suturing openings close to major bile ducts because of the risk of stricture. In the omentoplasty group, an omental flap with a preserved vascular supply was placed within the residual cavity. In the tube drainage group, a 26-Fr drain was placed inside the cavity. A closed-suction Jackson–Pratt drain was placed in Morison’s pouch in both groups.

Postoperative albendazole was administered at 10 mg/kg/day for three months in both groups according to the departmental protocol used during the study period, with treatment given during the first 20 days of each 30-day cycle followed by a 10-day treatment-free interval. These intervals were intended to reduce the potential risk of hepatotoxicity. Liver function tests were checked during the treatment-free interval of each cycle.

In both groups, surgical antibiotic prophylaxis consisted of piperacillin/tazobactam (4.5 g), administered 60 min before incision to provide coverage against Gram-negative organisms in this population at high risk of cystobiliary communication. Preoperative antibiotic treatment was individualized in patients presenting with cholangitis or an abscess. The choice and duration of therapeutic antibiotics were determined by infectious disease specialists according to existing cholangitis or abscess, infected cyst contents identified intraoperatively, postoperative signs of infection, and available culture results.

The same postoperative care approach was applied in both groups. Oral fluids were initiated 6–8 h after surgery in patients without gastrointestinal complications or contraindications to oral intake. Mobilization began during the same period with in-bed exercises in the intensive care unit and progressed to out-of-bed mobilization as tolerated after transfer to the surgical ward. An epidural catheter placed intraoperatively by the anesthesiology team was used for postoperative analgesia. Analgesic management was individualized according to analgesic requirements, renal function, and liver function tests. Supplemental paracetamol and/or dexketoprofen was administered as needed when clinically appropriate.

The primary outcome was a composite of postoperative bile leakage or a residual cavity-related complication occurring within the first 30 days after surgery. Residual cavity-related complications included cavity infection, abscess formation, and loculated fluid collections. Patients experiencing more than one component were counted only once in the composite outcome analysis. The individual components were also evaluated separately.

Secondary outcomes were operative time, length of hospital stay, surgical site infection, the need for percutaneous intervention within the first six postoperative months, and long-term recurrence. Postoperative cholangitis and overall postoperative morbidity were additionally evaluated as exploratory outcomes. Overall postoperative morbidity was defined as the occurrence of at least one episode of bile leakage, a residual cavity-related complication, surgical site infection, or postoperative cholangitis. Each patient was counted only once in this analysis.

Postoperative bile leakage was defined as macroscopic bilious fluid coming from the surgical drain, as determined by clinical assessment. Early ERCP was performed for leaks with a daily bile output > 100 mL, whereas low-output leaks were managed conservatively. Leaks that resolved within the first 10 postoperative days were classified as early postoperative bile leakage, whereas those persisting for more than 10 days were classified as biliary fistulas. This distinction constituted the clinical course classification used in the study. Biliary fistula was considered a subset of bile leakage and was not counted as an additional event in the composite outcome.

Patients with suspected postoperative complications underwent CT evaluation. Residual cavity infection was defined through a combined assessment of purulent drainage, a body temperature > 37.8 °C, a leukocyte count > 10,000/µL, and compatible imaging findings. Surgical site infections were assessed within the first 30 days after surgery according to the Centers for Disease Control and Prevention (CDC) definitions [12].

After surgical treatment and, where applicable, treatment of postoperative complications had been completed, patients were invited to attend an initial outpatient follow-up visit at one month. Subsequent visits were scheduled at three-month intervals during the first year and at six-month intervals thereafter. Follow-up included clinical assessment and routine complete blood count, biochemical tests, C-reactive protein measurement, and hepatobiliary US. CT was performed when additional cross-sectional imaging was recommended following US assessment. Long-term recurrence status was determined from the clinical and imaging findings obtained during follow-up. No patients were lost to follow-up.

Routine outpatient follow-up was performed by the surgical residents assigned to the outpatient clinic. Patients requiring hospitalization for postoperative complications were managed by their operating surgeons. Ward-level clinical data were collected by the ward resident, who was unaware of the patients’ study group assignments, and subsequently provided to the study coordinator. The coordinator, who was aware of treatment allocation, compiled these data into the study dataset. Statistical analyses were performed by a researcher who was not involved in the surgical procedures or data collection. The findings were interpreted jointly by the study coordinator, the researcher performing the statistical analyses, and the senior investigator.

### 2.3. Statistical Analysis

Statistical analyses were performed using R software (version 4.6.1; R Foundation for Statistical Computing, Vienna, Austria). Continuous variables were summarized as mean ± standard deviation and/or median [interquartile range], and categorical variables were presented as frequencies and percentages.

Age and operative time were compared between groups using Welch’s t test. Cyst size and length of hospital stay were compared using the Mann–Whitney U test. The Mann–Whitney U test was additionally applied to operative time as a sensitivity analysis. Binary categorical variables were compared using Fisher’s exact test, and variables with more than two categories were evaluated using the Fisher–Freeman–Halton exact test. A single overall comparison was performed for each multicategory variable, including presenting complaint, cyst location, and WHO stage.

The principal analysis of the primary composite outcome was based on event frequencies in the two groups and a two-sided Fisher’s exact test. Effect sizes were reported as the risk ratio and absolute risk difference for omentoplasty compared with tube drainage, with corresponding 95% confidence intervals. Confidence intervals for risk ratios were calculated using the logarithmic method. Confidence intervals for risk differences were calculated using the Newcombe method based on Wilson intervals without continuity correction [13].

Firth’s penalized likelihood logistic regression was used to further evaluate the association between residual cavity management and the primary composite outcome. This method was selected because of the limited sample size and sparse observations in some covariate combinations [14]. Models were fitted using the logistf package (version 1.26.1). A univariable model containing only the cavity management method was fitted first. An adjusted model was then constructed incorporating cavity management, cyst size, and cholangitis at presentation. Cyst size was entered as a continuous variable measured in centimeters. Preoperative cholangitis was defined as cholangitis documented at the initial presentation before biliary interventions.

Because of the substantial overlap between preoperative ERCP and cholangitis at presentation, ERCP was evaluated in an additional sensitivity model. This model included cavity management, cyst size, cholangitis at presentation, and preoperative ERCP. The adjusted models were additional exploratory analyses developed on clinical grounds after examination of the data. Covariates were not selected using univariable *p*-value screening or automated stepwise procedures.

Omentoplasty was the reference category in the regression models. Results were reported as odds ratios (ORs) with 95% profile penalized likelihood confidence intervals, and *p*-values were obtained from penalized likelihood ratio tests. Regression-derived ORs were reported separately from the risk ratios obtained in the unadjusted comparisons.

Unadjusted *p*-values were reported for the five secondary outcomes. To assess the impact of multiple comparisons, Holm adjustment was applied to operative time, length of hospital stay, surgical site infection, percutaneous intervention within the first six postoperative months, and long-term recurrence as an additional post hoc sensitivity analysis [15]. Individual components of the composite outcome and other exploratory comparisons were not included in this family of five secondary outcomes.

There were no missing data in the variables used for analysis; therefore, no imputation was performed, and all 60 patients were included in the analyses. All tests were two-sided, with a statistical significance threshold of 0.05.

Generative artificial intelligence was used to assist with regression model specification and preparation of the R analysis code. The researchers executed the code in R and checked the resulting outputs. The authors retained responsibility for the final model selection, interpretation, and reporting of the results.

## 3. Results

### 3.1. Patient and Cyst Characteristics

Sixty patients with HCE at high risk of biliary complications were included, with 30 patients in each group. The mean age was 43.2 ± 16.6 years in the omentoplasty group and 38.1 ± 15.4 years in the tube drainage group. Nineteen patients (63.3%) in the omentoplasty group and 17 (56.7%) in the tube drainage group were female. Right upper quadrant pain was the most common presenting complaint in both groups. No statistically significant between-group differences were observed in demographic characteristics, comorbidities, ASA class, clinical presentation, or preoperative procedures (Table 1).

A single cyst was present in 26 patients (86.7%) in the omentoplasty group and 22 (73.3%) in the tube drainage group. The median diameter of the dominant cyst was 10.0 [8.0–12.0] and 9.5 [8.0–12.0] cm, respectively. CE3 was the most frequently recorded WHO stage in both groups. No statistically significant between-group differences were observed in cyst number, dominant cyst diameter, WHO stage, or cyst location (Table 2).

### 3.2. Surgical Outcomes, Postoperative Complications, and Follow-Up

The primary outcome, comprising bile leakage and/or residual cavity complications, occurred in 6 patients (20.0%) in the omentoplasty group and 16 (53.3%) in the tube drainage group (*p* = 0.015). The risk ratio for omentoplasty relative to tube drainage was 0.375 (95% confidence interval [CI]: 0.170–0.826), and the absolute risk difference was −33.3 percentage points (95% CI: −52.8 to −8.9).

Bile leakage occurred in 6 patients (20.0%) and 11 patients (36.7%), respectively (*p* = 0.252), and residual cavity complications occurred in 2 (6.7%) and 8 (26.7%), respectively (*p* = 0.080). Surgical site infection was recorded in 1 patient (3.3%) in the omentoplasty group and 9 (30.0%) in the tube drainage group (*p* = 0.012). No patient in the omentoplasty group underwent percutaneous intervention within the first six months, compared with 8 patients (26.7%) in the tube drainage group (*p* = 0.005).

No hepatotoxicity was observed during albendazole treatment, and no unplanned treatment interruption or discontinuation due to hepatotoxicity was required.

The mean operative time was 131.3 ± 45.5 min in the omentoplasty group and 158.2 ± 50.1 min in the tube drainage group (*p* = 0.034). The median length of hospital stay was 8.5 [7.0–10.8] and 10.0 [8.0–13.0] days, respectively (*p* = 0.236). Overall postoperative morbidity was recorded in 7 patients (23.3%) and 17 patients (56.7%), respectively (*p* = 0.017). Surgical outcomes, postoperative complications, and follow-up data are presented in Table 3.

**Figure 2:**
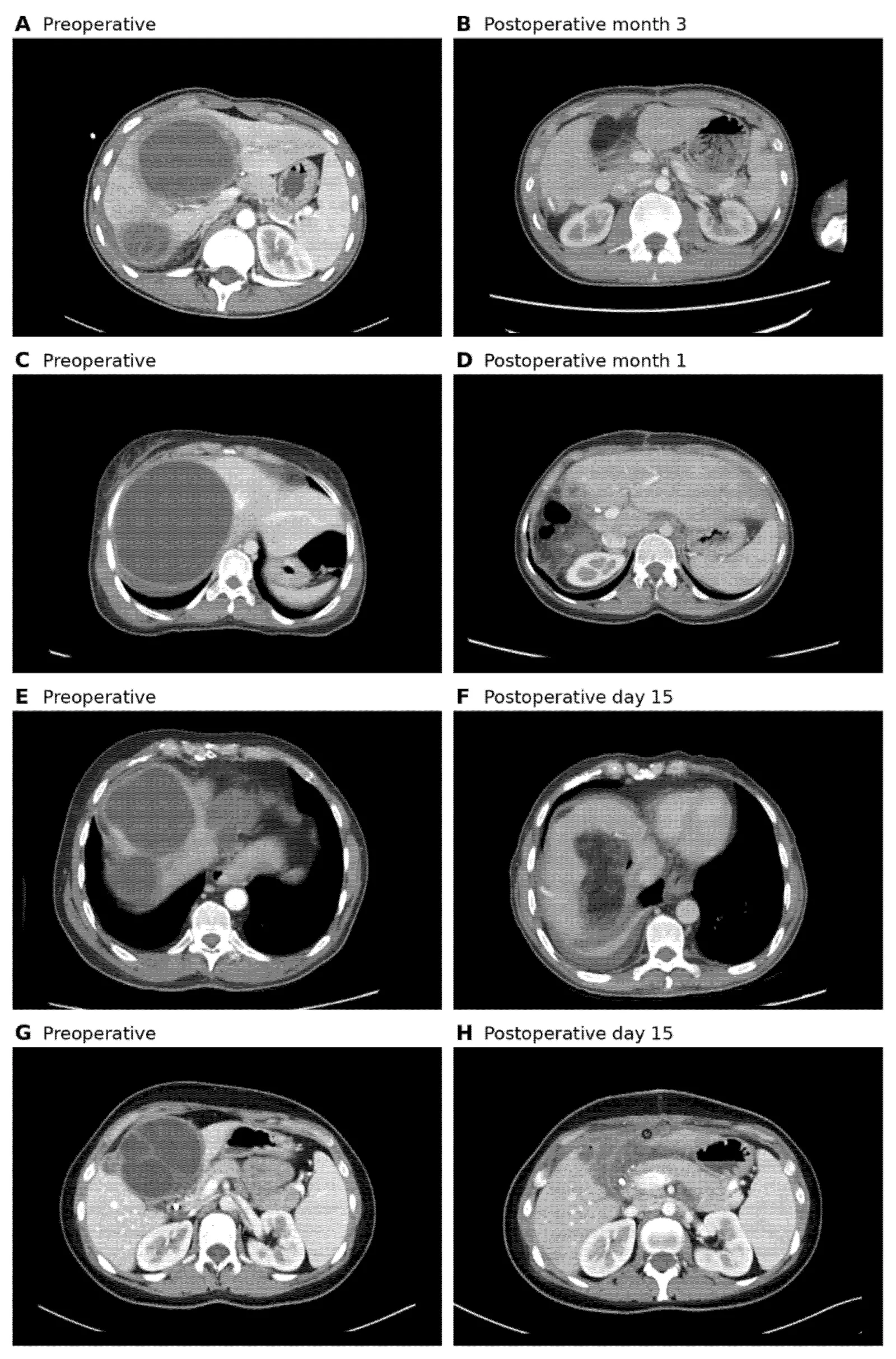
Preoperative and postoperative computed tomography images from four patients. Patients 1–3 underwent omentoplasty, and patient 4 underwent tube drainage. (**A**,**B**) Patient 1: preoperative and three-month postoperative images, respectively. (**C**,**D**) Patient 2: preoperative and one-month postoperative images, respectively. (**E**,**F**) Patient 3: preoperative and postoperative day 15 images, respectively. (**G**,**H**) Patient 4: preoperative and postoperative day 15 images, respectively.

Preoperative and postoperative CT images from four patients are presented in Figure 2.

The mean follow-up duration for the entire cohort was 68.8 ± 5.8 months, ranging from 60 to 78 months. All patients completed at least 60 months of follow-up, with no loss to follow-up. Recurrence was detected in 1 patient (3.3%) in the omentoplasty group and 4 (13.3%) in the tube drainage group (*p* = 0.353). The single recurrence in the omentoplasty group was local to the previous surgical site. Of the four recurrences in the tube drainage group, two were local to the previous surgical site, whereas two occurred elsewhere in the liver.

After Holm adjustment across the five secondary outcomes, the adjusted *p*-values were 0.049 for surgical site infection and 0.023 for percutaneous intervention. The adjusted *p*-value was 0.102 for operative time and 0.472 for both length of hospital stay and recurrence.

### 3.3. Regression and Sensitivity Analyses

All Firth logistic regression analyses of the primary outcome included 60 patients, of whom 22 experienced the primary outcome. In the univariable model, with omentoplasty as the reference, the odds ratio (OR) for tube drainage was 4.29 (95% CI: 1.46–13.87; *p* = 0.008).

In the model adjusted for cyst diameter and preoperative cholangitis, the OR for tube drainage was 5.25 (95% CI: 1.51–22.72; *p* = 0.008). In the same model, the OR was 14.15 for preoperative cholangitis (95% CI: 3.14–94.64; *p* <0.001) and 1.00 per 1 cm increase in cyst diameter (95% CI: 0.81–1.24; *p* = 0.972).

In the sensitivity model incorporating preoperative ERCP, the OR for tube drainage was 6.13 (95% CI: 1.72–27.02; *p* = 0.004). The OR was 50.58 for preoperative cholangitis (95% CI: 4.18–7229.99; *p* <0.001) and 0.17 for preoperative ERCP (95% CI: 0.0011–2.25; *p* = 0.198). Nine of the ten patients who underwent preoperative ERCP had preoperative cholangitis. The results of the three models are presented in Table 4.

Model 0 included residual cavity management alone. Model 1 included residual cavity management, cyst diameter, and preoperative cholangitis. Model 2 was the sensitivity model incorporating preoperative ERCP in addition to the variables in Model 1. All models included 60 patients and 22 primary outcome events. The adjusted models were specified after examination of the data.

Omentoplasty was the reference category for residual cavity management; the corresponding ORs express the odds for tube drainage relative to omentoplasty. Odds ratios and risk ratios are distinct measures. Confidence intervals were calculated using profile penalized likelihood, and *p*-values were obtained from penalized likelihood ratio tests. OR: odds ratio; CI: confidence interval; ERCP: endoscopic retrograde cholangiopancreatography.

In the additional sensitivity analysis of operative time using the Mann–Whitney U test, the *p*-value was 0.049.

## 4. Discussion

In this prospective study of patients with hepatic cystic echinococcosis at high risk of biliary complications undergoing conservative surgery, omentoplasty was associated with a lower incidence of the composite outcome than tube drainage. The primary outcome, comprising bile leakage and/or residual cavity complications, occurred in 6/30 patients (20.0%) in the omentoplasty group and 16/30 patients (53.3%) in the tube drainage group. The risk ratio was 0.375 (95% confidence interval: 0.170–0.826), with an absolute between-group difference of 33.3 percentage points. This association persisted after adjustment for cyst diameter and preoperative cholangitis and in the sensitivity analysis incorporating preoperative ERCP. These findings support the clinical importance of residual cavity management in postoperative morbidity in this patient population.

Our results are generally consistent with those of previous studies evaluating omentoplasty. Muftuoglu et al. reported that omentoplasty may reduce postoperative morbidity related to the residual cavity [4]. In the randomized study by Wani et al., the overall complication rate was lower in the omentoplasty group, although the difference did not reach statistical significance. Hospital stay was also shorter in the omentoplasty group. However, the exclusion of infected cysts should be considered when comparing their findings with those of our high-risk HCE cohort, which also included patients with existing or previously treated cyst-related complications [5]. The meta-analysis by Dziri et al. similarly reported fewer deep organ/space surgical site infections following omentoplasty after conservative HCE surgery [8]. Although this outcome differs from our composite endpoint, the direction of the findings supports a potential benefit of omentoplasty in reducing postoperative morbidity.

The systematic review and meta-analysis by Peng et al., which included various surgical procedures, provides further context. In the liver surgery subgroup, omentoplasty was associated with lower rates of overall complications, infection, and recurrence [9]. This broad assessment provides additional evidence of the potential benefits of omentoplasty in liver surgery. However, the inclusion of different surgical procedures and comparators limits direct extrapolation to patients with HCE at high risk of biliary complications.

A distinguishing feature of our study is its focus on patients with HCE at high risk of biliary complications requiring conservative surgery. Kayaalp et al. demonstrated that complicated cysts containing bile or pus were associated with more frequent postoperative biliary and cavity complications and longer hospital stays [10]. Their classification was based on cyst contents, whereas our cohort included both patients with existing or previously treated cyst-related complications and patients selected because of features indicating an increased risk of biliary complications. These populations should therefore not be considered equivalent. Our selection criteria provided a clinically focused comparison, but the cohort remained heterogeneous in terms of complication history, cyst characteristics, and the basis for classification as high risk.

The potential benefits of omentoplasty may be explained by the biological properties of the omentum. In addition to filling the residual cavity, its rich vascular and lymphatic networks and immunological properties may support local fluid clearance and tissue healing [6]. These characteristics provide a biological rationale for fewer cavity-related complications. However, these mechanisms were not directly investigated in our study, and the mechanisms underlying the observed clinical differences could not be established.

Both components of the composite outcome favored omentoplasty. Bile leakage occurred in 20.0% of patients in the omentoplasty group and 36.7% in the tube drainage group. These rates correspond to an absolute difference of 16.7 percentage points and an approximately 45.5% lower observed rate of bile leakage in the omentoplasty group. Although the difference did not reach statistical significance, its magnitude is clinically relevant and consistent with a potential benefit of omentoplasty. Residual cavity complications were also less frequent in the omentoplasty group (6.7% vs. 26.7%), although this difference was not statistically significant when analyzed separately. Thus, the principal finding is the lower incidence of the composite outcome. The differences in its individual components were in the same direction, but the magnitude of the association for each component requires evaluation in larger samples.

The adjusted analyses also supported the primary comparison. With omentoplasty as the reference, the odds ratio for the composite outcome associated with tube drainage was 5.25 (95% confidence interval: 1.51–22.72) in the model including cyst diameter and preoperative cholangitis, and 6.13 (95% confidence interval: 1.72–27.02) after the addition of preoperative ERCP. However, nine of the ten patients who underwent preoperative ERCP also had cholangitis, making it difficult to distinguish the separate contributions of these variables. The wide confidence intervals also indicate uncertainty in the estimates. Although Firth penalized logistic regression can reduce small-sample estimation bias and address separation, it does not eliminate confounding arising from nonrandomized group allocation [14]. These models, specified after examination of the data, should therefore be regarded as exploratory analyses supporting the association between omentoplasty and a lower incidence of complications.

The lower rates of surgical site infection and postoperative percutaneous intervention in the omentoplasty group are additional clinically relevant findings. These differences remained statistically significant after Holm adjustment across the five secondary outcomes. A lower requirement for percutaneous intervention is particularly relevant to the additional treatment burden experienced by patients after surgery. Considered alongside the composite outcome, these findings support the potential of omentoplasty to reduce postoperative morbidity.

Mean operative time was shorter in the omentoplasty group. Although mobilization of the omentum requires an additional surgical step, the procedures needed to position and securely fix an intracavitary drain during tube drainage may have contributed to the observed difference. Performance of all operations by the same hepatopancreatobiliary surgical team also supported consistency in technical practice. However, the duration of individual surgical steps was not recorded, and this explanation could not be directly verified. Furthermore, the difference in operative time did not remain statistically significant after Holm adjustment and should therefore be considered a supportive observation. Hospital stay was numerically shorter in the omentoplasty group, but no statistically significant between-group difference was demonstrated.

The minimum follow-up of 60 months for all patients, mean follow-up of 68.8 ± 5.8 months, and absence of loss to follow-up are important strengths of this study. This duration allowed assessment of late recurrence in addition to early postoperative complications. Recurrence occurred in one patient in the omentoplasty group and four patients in the tube drainage group. Despite this numerical difference favoring omentoplasty, the small number of events limits definitive assessment of the effect of either technique on recurrence. The observed difference in long-term recurrence therefore requires confirmation in larger series.

The prospective design, performance of surgery by the same team, and complete follow-up are major strengths. Nevertheless, the single-center setting and sample of 60 patients limit the generalizability of the findings. Because the study included only patients undergoing open conservative surgery, the findings cannot be directly generalized to laparoscopic procedures. The sample size calculation was based on the primary outcome, and statistical power may have been insufficient to detect differences in less frequent secondary outcomes. Alternating allocation did not provide randomization, and the predictability of the allocation sequence did not eliminate the possibility of selection bias. Despite adjustment, unmeasured clinical characteristics or factors not included in the models may have influenced the results. Although the resident collecting ward-level data was unaware of study group assignments, the coordinator compiling the dataset knew treatment allocation; therefore, the possibility of information bias cannot be excluded.

Outcome definitions and perioperative treatment should also be considered when interpreting the findings. Defining bile leakage by macroscopically bilious drainage limits comparability with studies using the ISGLS definition proposed for hepatobiliary surgery [16]. Although surgical antibiotic prophylaxis was the same in both groups, therapeutic antibiotic selection and duration were individualized according to clinical and microbiological findings. Differences in antibiotic exposure may therefore have influenced surgical site and residual cavity infection rates and represent a potential source of confounding when interpreting these outcomes. In addition, retrospective study registration and specification of the adjusted models after examination of the data underscore the need for confirmation in further studies.

## 5. Conclusions

In this prospective study, omentoplasty was associated with a lower incidence of the composite outcome than tube drainage in patients with HCE at high risk of biliary complications undergoing conservative surgery. This association persisted across the adjusted models examined. Lower rates of surgical site infection and postoperative percutaneous intervention further strengthen the clinical findings favoring omentoplasty. The approximately 45% lower observed rate of bile leakage also suggests a potential benefit, although the difference did not reach statistical significance when analyzed separately. These results support the use of omentoplasty for residual cavity management in appropriately selected patients. Its benefits and effect on long-term recurrence should be confirmed in larger, multicenter randomized trials.

## Figures and Tables

**Table 1:** Demographic and clinical characteristics.

Characteristic	Omentoplasty (*n* = 30)	Tube Drainage (*n* = 30)	*p* Value
Age, years	43.2 ± 16.6	38.1 ± 15.4	0.222
**Sex**			0.792
Female	19 (63.3)	17 (56.7)	—
Male	11 (36.7)	13 (43.3)	—
**Comorbidities**			
Cardiovascular disease	6 (20.0)	2 (6.7)	0.254
Hypertension	7 (23.3)	5 (16.7)	0.748
Diabetes mellitus	1 (3.3)	2 (6.7)	1.000
COPD	1 (3.3)	0 (0.0)	1.000
**ASA class**			0.554
I	21 (70.0)	23 (76.7)	—
II	7 (23.3)	7 (23.3)	—
III	2 (6.7)	0 (0.0)	—
**Clinical presentation**			0.583
Right upper quadrant pain	22 (73.3)	18 (60.0)	—
Diffuse abdominal pain	3 (10.0)	5 (16.7)	—
Cholangitis	5 (16.7)	7 (23.3)	—
**Preoperative procedures**			
ERCP	3 (10.0)	7 (23.3)	0.299
PAIR	2 (6.7)	1 (3.3)	1.000

Data are presented as *n* (%) or mean ± standard deviation. Welch’s *t*-test was used for age. Categorical variables were compared using Fisher’s exact test or the Fisher–Freeman–Halton exact test. For variables with multiple categories, the *p*-value refers to the overall distribution. Clinical presentation reflects the patient’s condition at the initial presentation. Previous PAIR refers to unsuccessful PAIR or PAIR complicated by abscess formation. ASA: American Society of Anesthesiologists; COPD: chronic obstructive pulmonary disease; ERCP: endoscopic retrograde cholangiopancreatography; PAIR: puncture, aspiration, injection, and reaspiration.

**Table 2:** Cyst characteristics.

Characteristic	Omentoplasty (*n* = 30)	Tube Drainage (*n* = 30)	*p* Value
**Number of cysts**			0.333
Single cyst	26 (86.7)	22 (73.3)	—
Two or more cysts	4 (13.3)	8 (26.7)	—
Dominant cyst diameter, cm	10.0 [8.0–12.0]	9.5 [8.0–12.0]	0.444
**WHO stage**			1.000
CE2	7 (23.3)	6 (20.0)	—
CE3 (a and b)	21 (70.0)	22 (73.3)	—
CE4	2 (6.7)	2 (6.7)	—
**Location of the dominant cyst**			0.491
Segment I	1 (3.3)	0 (0.0)	—
Segment II	4 (13.3)	0 (0.0)	—
Segment III	2 (6.7)	3 (10.0)	—
Segment IV	4 (13.3)	4 (13.3)	—
Segment V	2 (6.7)	5 (16.7)	—
Segment VI	4 (13.3)	3 (10.0)	—
Segment VII	3 (10.0)	4 (13.3)	—
Segment VIII	10 (33.3)	11 (36.7)	—

Data are presented as *n* (%) or median [first–third quartile]. Cyst diameter was compared using the Mann–Whitney U test. Categorical variables were compared using Fisher’s exact test or the Fisher–Freeman–Halton exact test. For categorical variables, the *p*-value refers to the overall distribution. In patients with multiple cysts, diameter, location, and WHO stage refer to the dominant cyst. WHO: World Health Organization; CE: cystic echinococcosis. The reported segment indicates the predominant location of the dominant cyst and does not imply that the cyst was confined to that segment.

**Table 3:** Surgical outcomes, postoperative complications, and follow-up.

Outcome	Omentoplasty (*n* = 30)	Tube Drainage (*n* = 30)	Unadjusted *p*	Holm-Adjusted *p*
**Primary outcome within 30 days**				
Bile leakage and/or residual cavity complications	6 (20.0)	16 (53.3)	0.015	—
**Components of the primary outcome**				
Bile leakage, including biliary fistula	6 (20.0)	11 (36.7)	0.252	—
Residual cavity complications	2 (6.7)	8 (26.7)	0.080	—
**Secondary outcomes**				
Operative time, minutes	131.3 ± 45.5	158.2 ± 50.1	0.034	0.102
Length of hospital stay, days	8.5 [7.0–10.8]	10.0 [8.0–13.0]	0.236	0.472
Surgical site infection	1 (3.3)	9 (30.0)	0.012	0.049
Percutaneous intervention within six months	0 (0.0)	8 (26.7)	0.005	0.023
Long-term recurrence	1 (3.3)	4 (13.3)	0.353	0.472
**Other postoperative findings**				
Biliary fistula	2 (6.7)	4 (13.3)	0.671	—
Postoperative ERCP	3 (10.0)	3 (10.0)	1.000	—
Postoperative cholangitis	1 (3.3)	2 (6.7)	1.000	—
Overall postoperative morbidity	7 (23.3)	17 (56.7)	0.017	—
**Follow-up**				
Follow-up duration, months	68.0 ± 6.1	69.7 ± 5.4	—	—

Data are presented as *n* (%), mean ± standard deviation, or median [first–third quartile]. Fisher’s exact test was used for categorical outcomes, Welch’s *t*-test for operative time, and the Mann–Whitney U test for length of hospital stay. Follow-up duration is presented descriptively. Holm adjustment was performed as an additional analysis across the five listed secondary outcomes only. A dash indicates that the corresponding comparison or adjustment was not performed. For the primary outcome, the risk ratio for omentoplasty relative to tube drainage was 0.375 (95% CI: 0.170–0.826), and the risk difference, calculated as omentoplasty minus tube drainage, was −33.3 percentage points (95% CI: −52.8 to −8.9). Confidence intervals were calculated using the logarithmic method for the risk ratio and the Newcombe method for the risk difference. Each patient was counted once in the primary outcome. Biliary fistula was a subset of bile leakage. Overall postoperative morbidity included patients with the primary outcome, surgical site infection, and/or postoperative cholangitis, with each patient counted once. ERCP: endoscopic retrograde cholangiopancreatography; CI: confidence interval.

**Table 4:** Firth logistic regression analyses for the primary composite outcome.

Model	Variable/Comparison	OR	95% CI	*p* Value
Model 0	Tube drainage vs. omentoplasty	4.29	1.46–13.87	0.008
Model 1	Tube drainage vs. omentoplasty	5.25	1.51–22.72	0.008
Model 1	Cyst diameter, per 1 cm increase	1.00	0.81–1.24	0.972
Model 1	Preoperative cholangitis, yes vs. no	14.15	3.14–94.64	<0.001
Model 2	Tube drainage vs. omentoplasty	6.13	1.72–27.02	0.004
Model 2	Cyst diameter, per 1 cm increase	1.01	0.82–1.25	0.901
Model 2	Preoperative cholangitis, yes vs. no	50.58	4.18–7229.99	<0.001
Model 2	Preoperative ERCP, yes vs. no	0.17	0.0011–2.25	0.198

## Data Availability

The datasets generated and analyzed during this study are available from the corresponding author upon reasonable request.
